# Reduced E-cadherin facilitates renal cell carcinoma progression by WNT/β-catenin signaling activation

**DOI:** 10.18632/oncotarget.15361

**Published:** 2017-02-15

**Authors:** Xinqi Zhang, Mingxi Yang, Hua Shi, Jianxin Hu, Yuanlin Wang, Zhaolin Sun, Shuxiong Xu

**Affiliations:** ^1^ Emergency Department, General Hospital of Jinan Military Area, Jinan, Shandong, 250031, China; ^2^ Department of Urology, Guizhou Provincial People's Hospital Affiliated to Guizhou Medical University, Guiyang, Guizhou, 550002, China

**Keywords:** renal cell carcinoma, E-cadherin, β-catenin, immunohistochemistry (IHC)

## Abstract

Reduced expression of E-cadherin was observed in renal cell carcinoma (RCC). However, its potential clinical value and correlation with WNT/β-catenin signaling in RCC progression was still unclear. Immunohistochemical staining was performed in RCC tissue microarray to examine the expression status and prognosis value of E-cadherin and β-catenin. The potential role of E-cadherin in β-catenin translocation was analyzed with immunobloting assays. A significant negative correlation was observed between E-cadherin and β-catenin expression in RCC tissues. E-cadherin inhibits β-catenin translocation from membrane to cytoplasm in RCC tissues, which was an important step for WNT/β-catenin signaling. Reduced E-cadherin expression was associated with poor prognosis. More importantly, E-cadherin-/β-catenin^+^ was an independent detrimental factor for survival estimation of RCC patients. Reduced E-cadherin expression in RCC promoted cancer progression via WNT/β-catenin signaling pathway activation. E-cadherin/β-catenin provides a valuable prognosis marker for RCC, which may be an effective target for RCC therapy.

## INTRODUCTION

Renal cell carcinoma (RCC) is one of the most common malignant tumor in urinary system with high mortality [1]. Increased incidence was observed during the last decades [2]. Nearly 40% of the RCC patients were died from cancer progression [3]. The prognosis markers are valuable for RCC patients. However, the exact mechanisms which promote the progression of RCC are still unclear.

Accumulated evidences supported that the deregulation of cell-to-cell adhesion molecules were involved in RCC progression, which promoted tumor cell invasion and metastatic activity [4, 5]. As a calcium-dependent cell adhesion molecule, E-cadherin maintains the integrity of epithelial cells by regulating actin cytoskeleton. Reduced E-cadherin expression or mutation induced function loss of the cadherin complex, which resulted in metastasis of epithelial malignancies [6–8]. However, the clinical value of E-cadherin in diagnosis and prognosis evaluation was still unclear in RCC.

As an important member of cadherin complex, β-catenin is involved in Ca^2+^-dependent, cell-to-cell adhesion maintains of epithelial cells [9]. The disruption of E-cadherin/β-catenin complex activates tumor metastasis in a series of epithelial malignancies [10–13]. Moreover, β-catenin works as a key factor of canonical WNT signaling pathway. Dysregulation of WNT/β-catenin signaling results in abnormal accumulation of β-catenin in cytoplasm of cancer cells, to develop a complex with a group of transcription factors [14, 15]. Thereby elevated canonical WNT signaling targeted genes are involved in carcinogenesis and progression [9, 16]. However, the clinical value of WNT signaling dysregulation in RCC is still not clear. Here, we focused on the deregulation of E-cadherin in RCC progression based on tissue microarray analysis, as well as its potential correlation with WNT/β-catenin signaling pathway.

## RESULTS

### E-cadherin expression and correlation with clinicopathological characteristics

**Figure 1:**
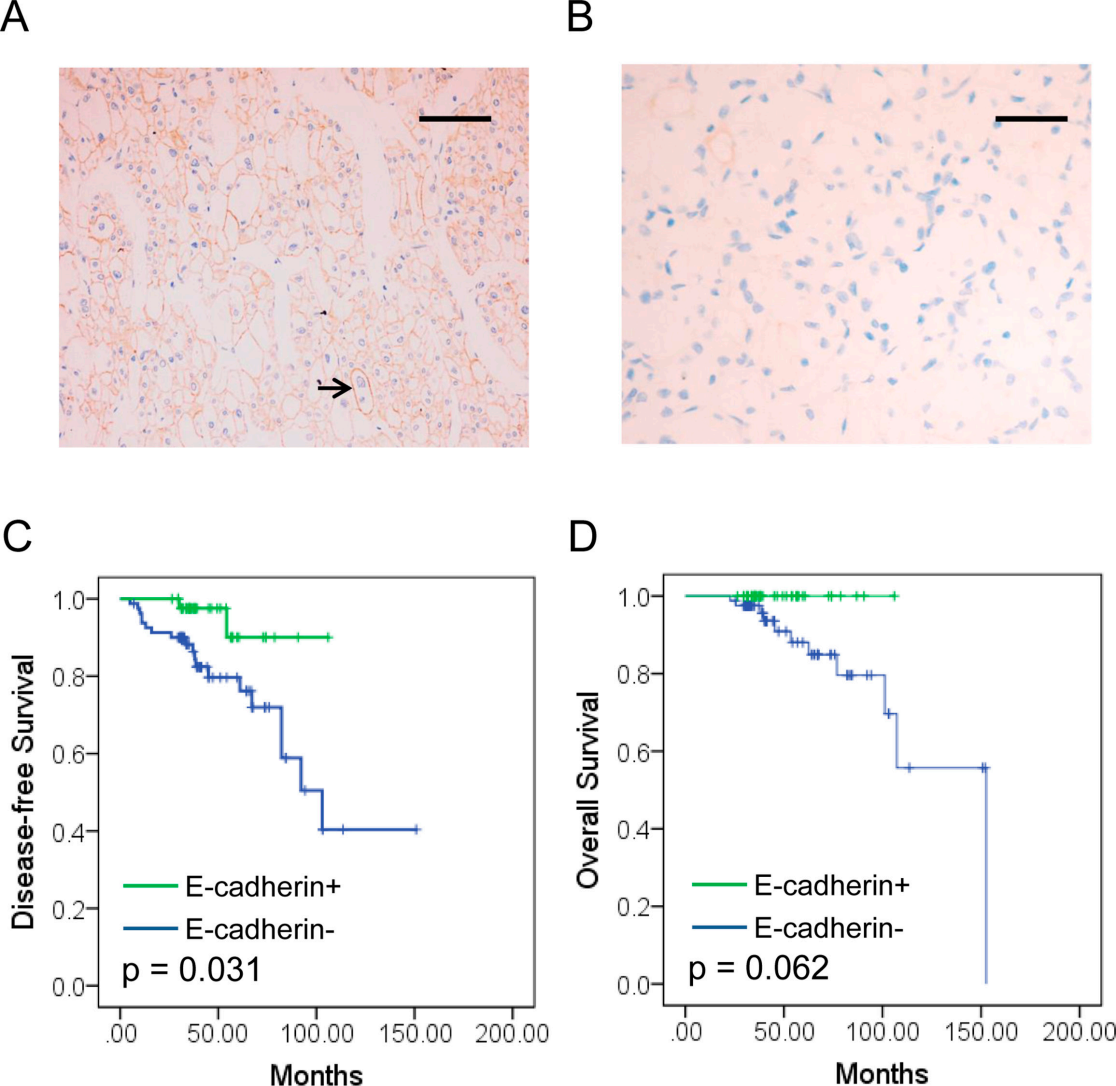
E-cadherin expression and prognosis value in RCC (**A**, **B**) E-cadherin expression images of RCC specimens. Positive E-cadherin expression was located on the membrane (A, black arrow) and negative E-cadherin expression image (B). Bar, 50 μm. (**C**, **D**) Kaplan-Meier analysis of disease-free survival (DFS, C) and Overall survival (OS, D) of RCC patients according to E-cadherin expression status.

IHC staining for E-cadherin was performed on TMA slides of RCCs. E-cadherin expression status was classified by the Allred score, score ≥ 4 was positive and score < 4 was negative. Stained E-cadherin protein was located on the cell membrane, which was arranged as brownish yellow granular or linear (Figure 1A). E-cadherin expression was observed in some renal tubules, but rarely found in nephron in the adjacent normal tissue (Supplementary Figure 1A). Significant decreased E-cadherin expression level was observed in the tumor tissue than adjacent normal tissue (Figure 1A and Supplementary Figure 1A). Moreover, lower positive percentage of E-cadherin expression was also observed in the tumor tissue (44/125) than adjacent normal tissue (106/125) (35.2% vs. 84.8%, χ^2^ = 48. 923, *P* < 0.001, Figure 1B).

Further analysis of E-cadherin expression and clinicopathological characteristics was also performed. Notably, significant correlation was observed in histological type (*p* < 0.001). Significant lower percentage of E-cadherin expression was observed in clear cell renal carcinoma (7/67) than other types (37/58) (10.45% vs. 63.79%). However, no significant correlation was observed between E-cadherin expression and age, gender, clinical stage, Fuhrman grade and necrosis (*P >* 0.05, Table 1).

**Table 1:** Correlations of E-cadherin and clinical characteristics

Variables	E-cadherin Expression		*p Value
	High	Low	
Gender			
Male	25	58	0.095
Female	19	23	
Age (years)			
> 56	24	38	0.415
≤ 56	20	43	
Surgical procedure			
Partial nephrectomy	10	14	0.461
Radical nephrectomy	34	67	
Tumor size, cm			
< 5.25	25	38	0.290
> 5.25	19	43	
TNM stage			
I–II	32	61	0.752
III–IV	12	20	
Histological subtype			
Clear cell	7	60	< 0.001
Papillary	16	12	
Chromophobe	18	1	
Others	3	8	
Necrosis			
Yes	11	23	0.684
No	33	58	
Fuhrman grade			
G1–2	25	46	0.998
G3–4	19	35	

* *P* value was estimated with Chi-square test.

### Prognosis value of E-cadherin expression in RCC patients

Kaplan-Meier (KM) analyses for disease-free survival (DFS) and overall survival (OS) were performed to investigate the prognostic value of E-cadherin expression in RCC. All the patients involved in our study were followed-up and the median period of 52.27 months (range: 22.8–152.6 months). Disease progression was observed in 21 patients (16.80%) during the period. Distant metastasis was observed with 95.24% of disease-progressed patients (20/21). The survival analysis revealed that E-cadherin^−^ patients showed no significant difference in overall survival estimation (OS) compared to other patients (*P* = 0.062. Figure 1C). However, significant benefit was observed for those patients with RCC positive for E-cadherin (*P* = 0.031. Figure 1D). To evaluate the independent prognostic significance of E-cadherin expression, a univariate analysis was performed with E-cadherin status (Table 2). The results showed that E-cadherin negative expression was a significant detrimental factor for DFS (HR = 0.229, *P* = 0.049), but not for OS (HR = 0.028, *P* = 0.266, Table 2). Furthermore, multivariate analyses were also performed with E-cadherin expression status. Factors associated with prognosis of RCC were included, such as age, gender, tumor size, tumor stage, Fuhrman grade and necrosis. The result indicated that E-cadherin expression was a beneficial factor for patients’ DFS (HR = 0.0.206, 95% CI: 0.046–0.928, *P* = 0.040). However, no significant correlation was observed with OS (HR *=* 0.000, 95% CI: 0.000–4.007E205, *P* = 0.960. Table 3). In addition, higher age had positive impact on both DFS (HR = 0.281, 95% CI: 0.101–0.786, *P* = 0.016) and OS (HR *=* 0.149, 95% CI: 0.028–0.786, *P* = 0.025. Table 3). Our data suggested that reduced E-cadherin expression was correlated with progression of RCC.

**Table 2:** Univariate and multivariate analyses of E-cadherin expression in disease-free survival and overall survival

Variable analysis	Disease-Free Survival			Overall Survival		
	HR	95% CI	*p*	HR	95% CI	*p*
Univariate	*N* = 125			*N* = 125		
E-cadherin	0.229	0.053–0.991	0.049	0.028	0.001–15.090	0.266
Multivariate	*N* = 125			*N* = 125		
Gender	0.749	0.279–2.008	0.565	0.541	0.111–2.637	0.447
Age	0.281	0.101–0.786	0.016	0.149	0.028–0.786	0.025
Size	0.708	0.209–2.400	0.579	0.505	0.061–4.207	0.528
Stage	1.779	1.007–3.142	0.047	2.165	0.951–4.930	0.066
Necrosis	2.346	0.734–7.500	0.150	1.298	0.250–6.750	0.756
Fuhrman	1.249	0.736–2.118	0.409	1.868	0.841–4.150	0.125
E-cadherin	0.206	0.046–0.928	0.040	0.000	0.000–4.007E205	0.960

The variables were compared in the following ways: Age, ≥ 57 years vs. < 57 years; Gender, male vs. female; E-cadherin expression, positive vs. negative; size, > 5.25 vs. < 5.25; Stage, III–IV vs. I–II; Necrosis, yes vs. no; Fuhrman grade, G3-4 vs. G1-2.

**Table 3:** Correlations of β-catenin and clinical characteristics

Variables	β-catenin expression		*P* Value
	High	Low	
Gender			
Male	63	20	0.103
Female	26	16	
Age (years)			
> 56	42	20	0.397
≤ 56	47	16	
Surgical procedure			
Partial nephrectomy	14	10	0.121
Radical nephrectomy	75	26	
Tumor size, cm			
< 5.25	41	22	0.128
> 5.25	48	14	
TNM stage			
I–II	69	24	0.208
III–IV	20	12	
Histological subtype			
Clear cell	56	11	0.222
Papillary	9	19	
Chromophobe	17	2	
Others	7	4	
Necrosis			
Yes	28	6	0.092
No	61	30	
Fuhrman grade			
G1–2	45	26	0.030
G3–4	44	10	

* *P* value was estimated with Chi-square test.

### E-cadherin expression correlated with WNT/β-catenin signaling

**Figure 2:**
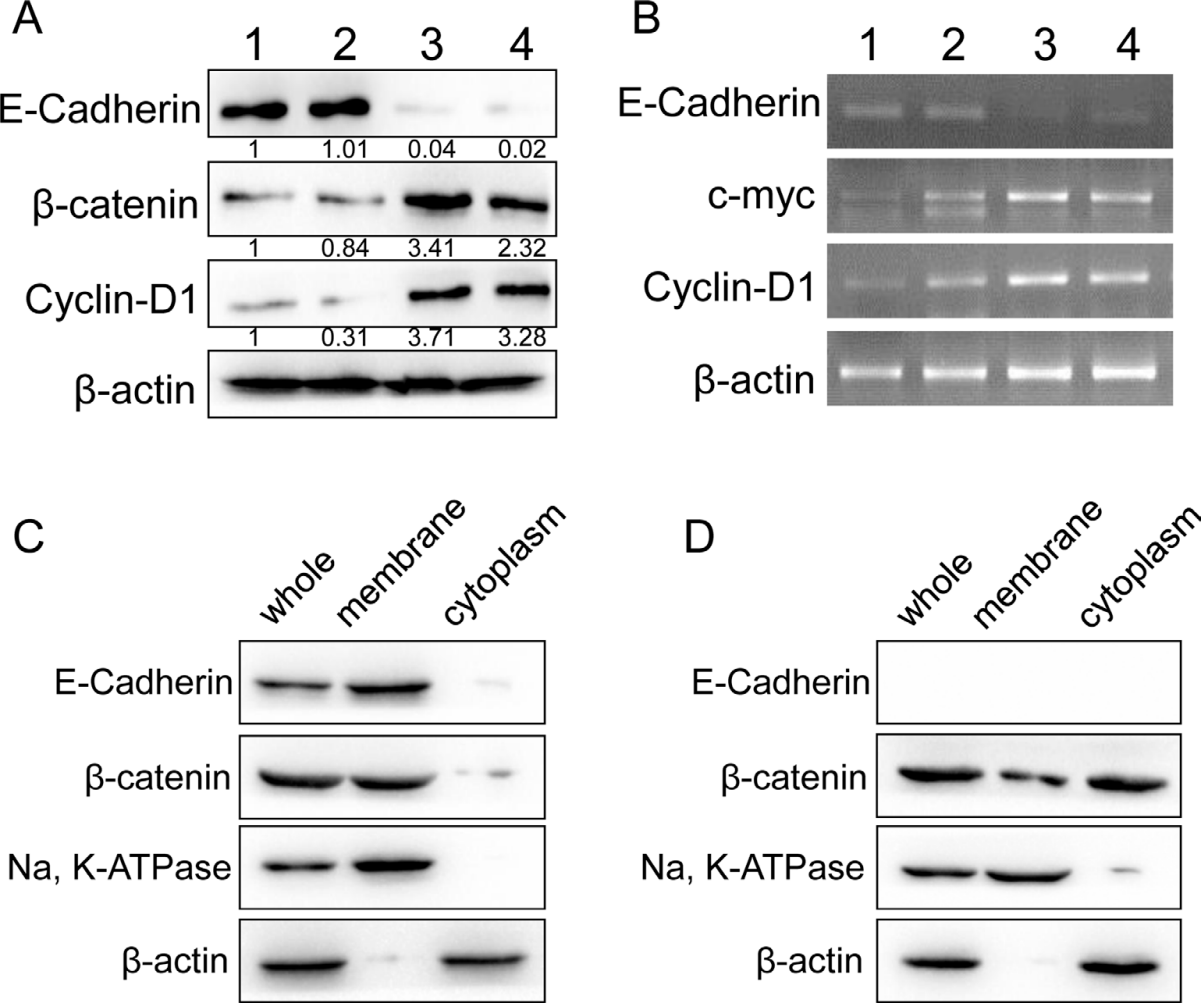
Correlation between E-cadherin and WNT/β-catenin signaling in RCC tissues (**A**) E-cadherin, β-catenin and cyclin D1 protein levels were analyzed with western blot in four fresh RCC tissues. β-actin was used as loading control. (**B**) mRNA levels of E-cadherin and WNT/β-catenin signaling targeted genes was studied with RT-PCR in the four fresh RCC tissues. β-actin was used as control. (**C**, **D**) β-catenin location in E-cadherin positive (C) or negative (D) specimens were studied with immunobloting assays. Na, K ATPase was used as membrane control, and β-actin as cytoplasm control. Representative images from at least three independent experiments are shown.

To investigate the underlie mechanism of E-cadherin participated RCC progression, Western blot assays for WNT/β-catenin signaling was performed in four fresh clinical specimens. Increased WNT/β-catenin signaling activation was observed in E-cadherin negative specimens, whereas inhibited WNT/β-catenin signaling in E-cadherin positive ones (Figure 2A). Further RT-PCR assays were performed with clinical specimens for Wnt/β-catenin targeted genes, including c-myc and cyclin D1. Elevated expression of Wnt/β-catenin targeted genes was shown in E-cadherin negative specimens (Figure 2A and 2B). To further investigate the correlation of E-cadherin and Wnt/β-catenin signaling, we carried out immunobloting assays to investigate the translocation of β-catenin in fresh RCC tissues. We found that β-catenin was concentrated in the membrane in E-cadherin positive specimens (Figure 2C). Increased β-catenin translocation for membrane to cytoplasm was observed in E-cadherin negative speicimes than E-cadherin positive ones (Figure 2D). Our results suggested a potential role of WNT/β-catenin signaling in the progression of E-cadherin negative RCC.

### β-catenin expression and correlations with E-cadherin in RCC

**Figure 3:**
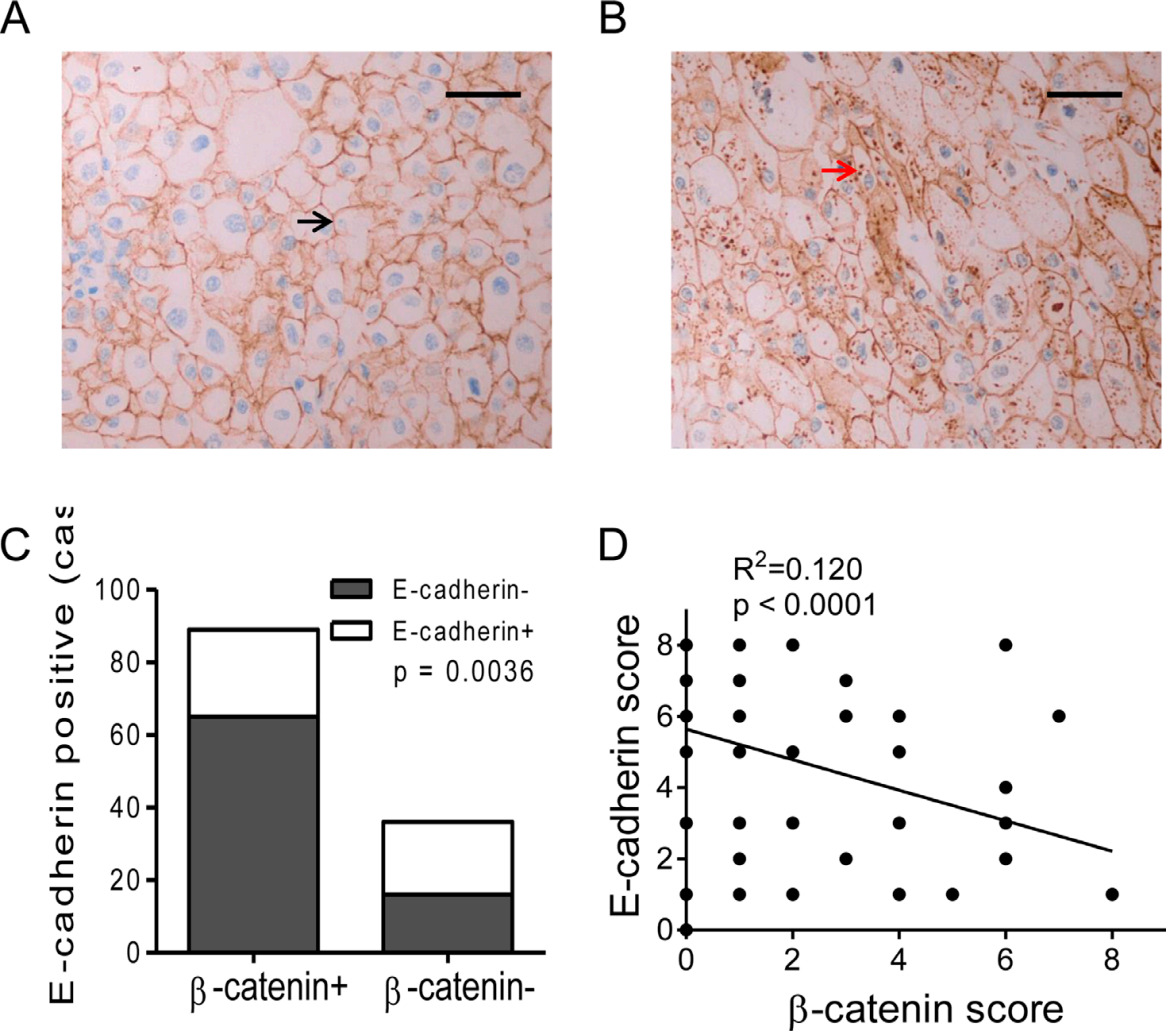
Expression of β-catenin and correlation with E-cadherin in RCC specimens (**A**, **B**) β-catenin expression images of RCC specimens. Positive β-catenin expression was mainly located on the membrane in E-cadherin positive specimen (A, black arrow), and cytoplasmic accumulation of β-catenin in E-cadherin negative specimen (B, red arrow). Bar, 50 μm. (**C**) The percentage of E-cadherin+ was compared between β-catenin positive or negative RCCs. (**D**) Negative correlation of E-cadherin and β-catenin expression scores in RCC specimens according to a linear regression.

Another IHC staining for β-catenin was performed with TMA specimens of RCC. Positive staining of β-catenin was located in cell membrane/cytoplasm (Figure 3A, 3B and Supplementary Figure 1B). More importantly, β-catenin expression in RCC cells was decreased or disappeared in membrane (Figure 3A), whereas abnormal accumulation in cytoplasm (Figure 3B). About 71.2% of renal cell carcinoma specimens were positive for β-catenin (89/125, Figure 3C). These results provided further evidence of the translocation of β-catenin from membrane to cytoplasm in RCC. Further analysis for the correlation of E-cadherin and β-catenin expression was performed. Lower percentage of E-cadherin expression was observed in β-catenin positive specimens than negative ones (26.97% vs. 55.56%, *P* < 0.01, Figure 3C and Supplementary Figure 2A, 2B). Moreover, significant negative correlation was observed between β-catenin scores and E-cadherin scores according to a linear regression analyze (*R*^2^ = 0.120, *P* < 0.0001, Figure 3D). Our results suggested a negative correlation between β-catenin and E-cadherin expression in RCC.

### Correlations between β-catenin and clinicopathological characteristics

In RCC specimens, significantly increased β-catenin expression was observed in higher Fuhrman grade tissues than lower ones (Fuhrman grade 3–4 vs. 1–2, *P* = 0.030, Table 3). However, no significant correlation was observed in β-catenin expression and other variables, including age, gender, tumor size, clinical stage, histological type and necrosis (*P >* 0.05, Table 3). Our results suggested that the activated WNT/β-catenin signaling was a potential detrimental actor for RCC patients.

### Prognostic value of E-cadherin/β-catenin expression in RCC

**Figure 4:**
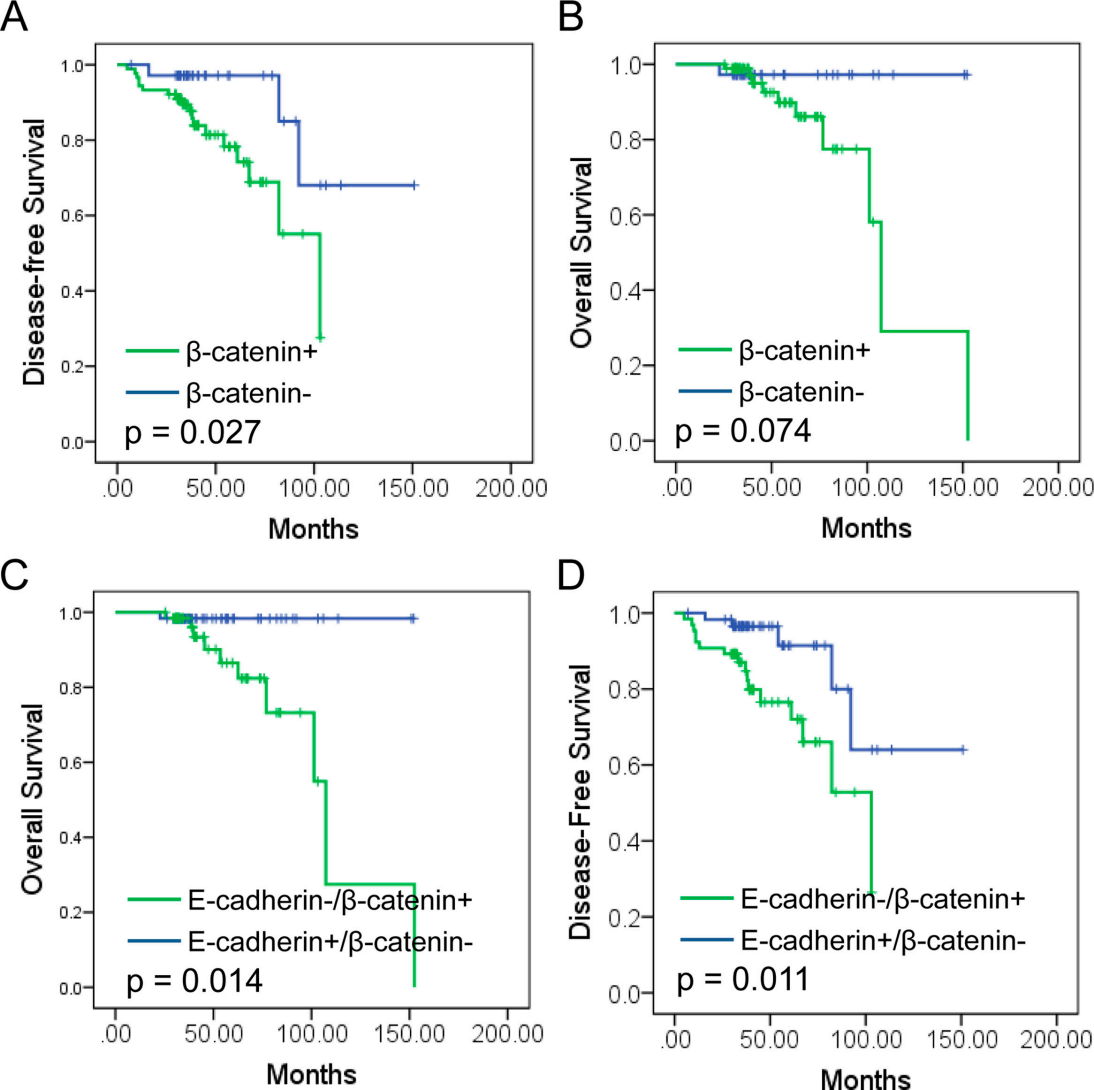
Prognostic value of E-cadherin and β-catenin expression in RCC (**A**, **B**) Kaplan-Meier analysis of β-catenin in disease-free survival (DFS, A) and overall survival (OS, B) in RCC patients. (**C**, **D**). Kaplan-Meier analysis of E-cadherin/β-catenin in disease-free survival (DFS, C) and overall survival (OS, D) in RCC patients.

Further study was performed to study the prognostic value of β-catenin expression in RCC. Significant worse DFS was observed in β-catenin positive patients than negative ones (*P* = 0.027, Figure 4A). However, no significant difference was observed in OS between two groups (*P* = 0.074, Figure 4B). Further survival analysis revealed that E-cadherin^−^/β-catenin^+^ patients showed significant worse prognosis in both OS and DFS compared to other patients (*P* = 0.014, *p* = 0.011. Figure 4C, 4D).

To evaluate the independent prognostic significance of E-cadherin^−^/β-catenin^+^, a univariate analysis was performed with E-cadherin^−^/ β-catenin^+^ (Table 4). The results showed that E-cadherin^−^/β-catenin^+^ expression was a significant detrimental factor for DFS (HR = 3.433, 95% CI: 1.249–9.433, *P* = 0.017) and OS (HR = 8.761, 95% CI: 1.103–69.556, *P* = 0.0.040. Table 4). Furthermore, multivariate analyses were performed with combined expression status of E-cadherin-/β-catenin+. Our results indicated that E-cadherin-/β-catenin+ co-expression was significantly associated with patients’ poor DFS (HR = 4.105, 95% CI: 1.329–12.681, *P* = 0.030), but not associated with OS (HR = 4.006, 95% CI: 0.903–68.373, *P* = 0.222. Table 4). In addition, higher tumor stage had negative impact on DFS (HR = 1.891, 95% CI: 1.036–3.454, *P* = 0.038. Table 4).

**Table 4:** Univariate and multivariate analyses of β-catenin expression in disease-free survival and overall survival

Variable analysis	Disease-Free Survival			Overall Survival		
	HR	95% CI	*p*	HR	95% CI	*p*
Univariate	*N* = 125			*N* = 125		
E-cad-/β-cat+	3.433	1.249–9.433	0.017	8.761	1.103–69.556	0.040
Multivariate	*N* = 125			*N* = 125		
Gender	1.029	0.371–2.857	0.956	0.691	0.119–3.473	0.668
Age	0.371	0.137–1.003	0.051	0.220	0.048–1.340	0.071
Size	0.620	0.171–2.240	0.465	0.709	0.069–5.115	0.733
Stage	1.891	1.036–3.454	0.038	1.903	0.874–4.653	0.129
Necrosis	2.195	0.691–6.977	0.183	0.954	0.212–5.586	0.956
Fuhrman	1.236	0.739–2.066	0.420	1.845	0.880–4.514	0.150
E-cad-/β-cat+	4.105	1.329–12.681	0.030	4.006	0.903–68.373	0.222

CI = confidence interval; HR = hazard ratios.

The variables were compared in the following ways: Age, ≥ 57 years vs. < 57 years; Gender, male vs. female; β-catenin expression, positive vs. negative; size, > 5.25 vs. < 5.25; Stage, III–IV vs. I–II; Necrosis, yes vs. no; Fuhrman grade, G3-4 vs. G1-2.

## DISCUSSION

The complex of E-cadherin/β-catenin attaches to actin cytoskeleton to regulate cell adhesion [17]. Loss function of E-cadherin complex will result in reduced cell adhesion and elevated tumor migration [18–21]. Combined allelic loss (16q) and point mutation in the remaining allele, as well as aberrant methylation of CpG islands in its promoter, result in reduced E-cadherin expression in malignances [22]. More importantly, increased E-cadherin expression in RCC cells inhibits tumor growth and prolongs survival *in vitro* [23]. Although the significance of E-cadherin in malignant biological behavior is well analyzed, the clinical value of E-cadherin expression in RCC is still under investigation. In this study, we observed reduced or loss of E-cadherin protein expression in the majority of examined RCC tissue microarray, especially clear cell renal carcinoma. More importantly, reduced E-cadherin expression was a significant detrimental factor for the prognosis of RCC. Increased disease progression was observed in the patients with E-cadherin negative RCCs. Moreover, significant reduced E-cadherin expression and increased metastasis were observed in clear cell renal cell carcinoma. Further study will be performed for the potential basic mechanism and clinical value.

A variety of signaling pathways have already shown to be involved in carcinogenesis and progression of RCC, including PI3K/Akt/mTOR, HGF/Met and VHL/hypoxia cellular signaling [24–27]. Recent studies have also shown aberrant signal transduction of WNT/β-catenin is correlated with an aggressive phenotype [15, 28, 29]. However, the exact mechanisem of WNT/β-catenin classical pathway involved in RCC progression is still under investigation. Interestingly, previous studies and our study declared that WNT/β-catenin pathway is deregulated especially in poorly differentiated and late-staged RCCs [30, 31]. E-cadherin shows a clear membranous staining pattern, whereas β-catenin shows a cytoplasmic and membranous localization. Our study indicated that accumulated β-catenin cytoplasmic expression in the absence of E-cadherin expression in RCC tissues, which was correlated with tumor invasiveness and poor prognosis. We provided further evidence that accumulation of β-catenin was associated with advanced Fuhrman grade. Additionally, we showed that more renal clear cell carcinomas exhibited increased β-catenin expression, which was just the opposite of E-cadherin.

In the current study, we co-analyzed the clinical value of E-cadherin/β-catenin protein complex in RCC specimens. Importantly, the cytoplasmic accumulation of β-catenin protein and reduced expression of E-cadherin were associated with an aggressive biological behavior of RCCs [32, 33]. Worse prognosis was estimated for the patients with E-cadherin^−^/β-catenin^+^ RCCs, which further support that WNT/β-catenin classical signaling participated in the progression of RCCs. This event potentially explains the altered intracellular modification of the cytoskeleton protein complexes involving cadherins and the actin binding proteins during epithelial malignant progression [34, 35]. This genetic imbalance releases the metastatic process due to disruption of cell-to-cell adhesion. However, further mechanism analysis of E-cadherin and WNT/β-catenin in RCCs should be studied in the future, and preclinical studies for potential therapy targets in this signaling should be performed to improve treatment response.

In conclusion, aberrant expression of E-cadherin/β-catenin in RCCs participates in metastatic progression. Based on the corresponding gene deregulation mechanisms, there is an increasing need for developing and evaluating inhibitory targets and agents for RCC therapy [36–38]. The expression status of E-cadherin/β-catenin should be paid attention in the clinical treatment of RCCs.

## MATERIALS AND METHODS

### Patients and samples

Totally 125 RCC patients were involved in our study. All patients were diagnosed as renal cell carcinomas and underwent surgery between 2003 and 2010 at General Hospital of Jinan Military Region, Shandong, China. Tissue microarray was prepared with collected tumor specimens. Clinical and pathological information was collected including age, gender, tumor size, TNM stage, histological type, necrosis and Fuhrman grade. All patients were followed-up every 6 months, which was carried out by telephone interview, outpatient records or death certificate review. Fresh tissues of renal cell carcinoma were also collected for immunoblotting assays and reverse transcription PCR. Our study was approved by the Ethics Committee of General Hospital of Jinan Military Region. Informed consent was accepted by the patients according to the research proposals.

### Tissue microarray (TMA) and immunohistochemical staining (IHC)

Hematoxylin- and eosin- (H&E) staining of each case was performed to confirm diagnosis and select representative area. TMA was established with representative areas of each tumor with a diameter of 2.0 mm from formalin-fixed, paraffin-embedded tissue blocks. TMA sections were prepared at 5μm of thickness for IHC staining according the following steps: deparaffinized in xylene, hydrated with gradient ethanol, antigen retrieval with 0.01 M sodium citrate for 17 minutes and immersed in 3% H_2_O_2_, incubated with 10% goat serum. Primary antibodies against human E-cadherin and β-catenin (GeneTex, San Antonio, TX) were incubated respectively at 4°C overnight. Then the sections were stained with peroxidase-conjugated avidin, followed with 3,3-diaminobenzidine tetrahydrochloride (DAB) and hematoxylin blue was used to counterstain. Assigned IHC positive RCC tissues, whose expression were confirmed by Western blot assays, were stained as positive control. Control IgG with equal concentration was used as negative control in each stain.

### IHC evaluation

The evaluation of E-cadherin and β-catenin levels was performed with Allred scoring system [39]. Brown membrane staining for E-cadherin and brown membrane and cytoplasm staining for β-catenin were considered as positive staining. The score for each section was determined by staining intensity and percentage of stained cells. Staining intensity score was arranged into: 0 (no staining), 1 (weak staining), 2 (moderate staining) and 3 (strong staining). Positive percentage score was arranged into: 0 (none); 1 (< 1%); 2 (1% to 10%); 3 (10% to 35%); 4 (35% to 70%); and 5 (> 70%). The sum of intensity score and percentage score was the final score of each case. The score ≥ 4 was positive and < 4 was negative with a statistical analysis. IHC evaluation was performed by two pathologists in a blinded manner.

### Western blot analysis

Fresh RCC tissues were collected and homogenized with lysis buffer, which contained 50 mM Tris-HCl, 150 mM NaCl, 1 mM EDTA, 1 mM phenylmethylsulfonyl fluoride, 1% Triton X-100, 0.1% SDS, and 0.1% sodium deoxycholate, pH 7.2. The homogenates were centrifuged at 1000 g for 10 min at 4°C. The membrane and cytosol fractions were prepared with Membrane and Cytosol Protein Extraction Kit according to the manufacturer's instructions (Beyotime Institute of Biotechnology, China). Protein concentration of the supernatant was measured by the Bradford method. Equal amounts of proteins (20 μg) of each sample were separated on 9% SDS-PAGE and electroblotted on polyvinylidene fluoride membranes. Blocked membranes were incubated overnight at 4°C with antibodies against E-cadherin, β-catenin cyclinD1 (GeneTex, San Antonio, TX), Na, K-ATPase (Abcam Inc., Cambridge, MA) and β-actin (Cell Signaling Technology, Beverly, MA) , respectively. The membranes were probed respectively with HRP-conjugated secondary antibodies (1:4,000) at room temperature for 1 h. Immunoreactive protein bands were visualized by use of an enhanced chemiluminescence detection system (GE Biosciences, Buckinghamshire, England) according to the manufacturer's protocol.

### Reverse transcription PCR (RT-PCR)

Total cellular mRNA was extracted from fresh RCC tissues using RNAiso Plus Reagent (TaKaRa, Japan) and reverse-transcribed with 1st Strand cDNA Synthesis Kit (TaKaRa, Japan) according to the introduction. The RNA concentration was determined by Thermo Scientific NanoDrop 2000 detector. Total of 5 μg mRNA was used for RT-PCR. Primers for E-cadherin, cyclinD1, c-myc and β-actin used in our study were listed as below: CDH1-forward: AAAGGCCCATTTCCTAAAAACCT; Reverse: TGCGTTCTCTATCCAGAGGCT. CCND1-forward: CAA TGACCCCGCACGATTTC; Reverse: CATGGAGGGC GGATTGGAA. MYC-forward: CACACCCACAATTC AGGAAGAG; Reverse: GACGTGCTACAAGGTGGCA. ACTB-forward: TTTTGGCTATACCCTACTGGCA; Reverse: CTGCACAGTCGTCAGCATATC. RT-PCR was performed with a One Step RT-PCR Kit Ver.2 (TaKaRa, Japan). Each quantity of mRNA expression was corrected by corresponding β-actin mRNA expression.

### Statistical analysis

The relationship between E-cadherin and β-catenin expression and clinicopathologic parameters were analyzed using a two- tailed Chi-square test or Fisher's exact test. The correlation between E-cadherin and β-catenin expression status was analyzed with the Spearman's rank test. Survival estimation was performed with the Kaplan-Meier method and log rank test. Univariate or multivariate analysis of prognostic factors was analyzed with Cox proportional hazards regression models. All statistical analyses were performed using the SPSS software system (version 19.0; SPSS, Inc., Chicago, IL, USA). *P* < 0.05 was considered to be statistically significant.

## SUPPLEMENTARY MATERIALS FIGURES


