# Supplementary material for: Reduced E-cadherin facilitates renal cell carcinoma progression by WNT/β-catenin signaling activation
Source: Oncotarget. 2017 Feb 15;8(12):19566–76. doi: 10.18632/oncotarget.15361 (PMC5386706; doi:10.18632/oncotarget.15361)
Supplement: Supplementary file 1 [file oncotarget-08-19566-s001.pdf]

Reduced E-cadherin facilitates renal cell carcinoma progression by WNT/β-catenin signaling activation

Supplementary Materials

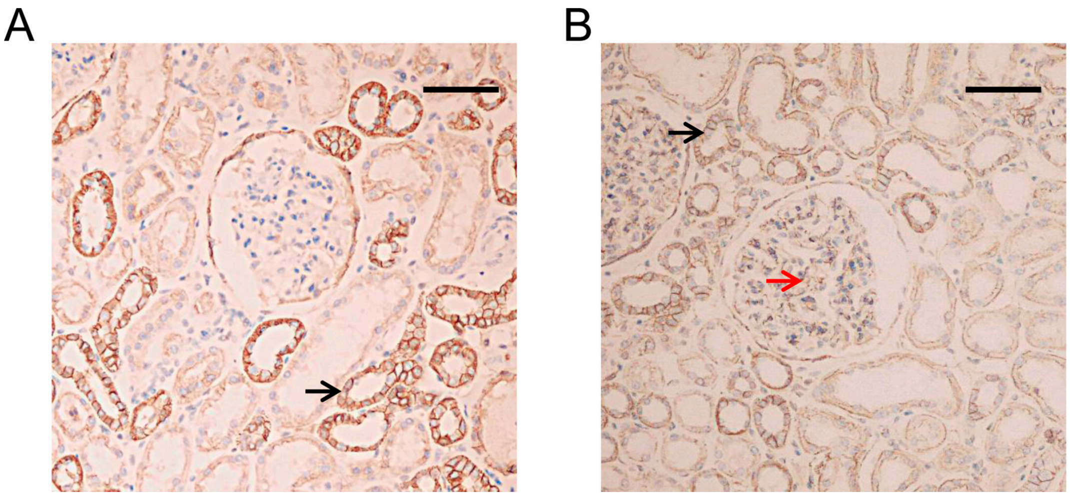

Supplemental Figure 1: E-cadherin and β-catenin expression in adjacent normal renal tissues. E-cadherin expression was observed in some renal tubules (**A**, black arrow), but rarely found in nephron. Weak β-catenin expression was observed in renal tubules (**B**, black arrow) and nephron (B, red arrow). Bar, 50 µm.

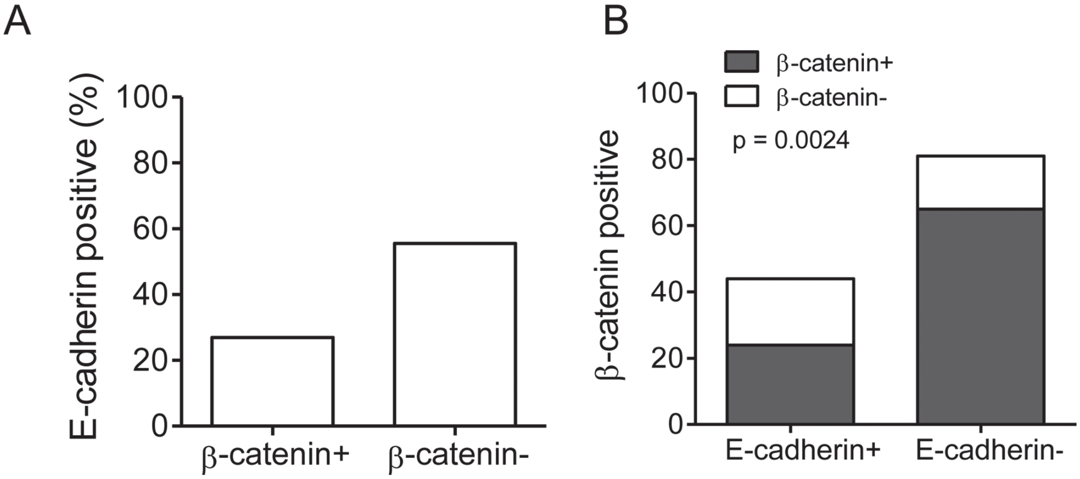

Supplemental Figure 2: The correlation of E-cadherin and β-catenin expression in renal cell carcinoma. (**A**) The comparison of E-cadherin$^+$ percentage between β-catenin positive specimens than negative ones (26.97% vs. 55.56%). (**B**) The comparison of β-catenin$^+$ cases in E-cadherin positive or negative RCCs. $P = 0.0024$.
